# Modeling transmission dynamics of severe acute respiratory syndrome coronavirus 2 in São Paulo, Brazil

**DOI:** 10.1590/0037-8682-0553-2020

**Published:** 2021-01-29

**Authors:** Pedro Alexandre da Cruz, Leandra Cristina Crema-Cruz, Fabrício Souza Campos

**Affiliations:** 1 Universidade Federal do Tocantins, Colegiado de Ciências Exatas e Biotecnológicas, Campus de Gurupi, Gurupi, TO, Brasil.; 2 Universidade Federal do Tocantins, Laboratório de Bioinformática e Biotecnologia, Campus de Gurupi, Gurupi, TO, Brasil

**Keywords:** SARS-CoV-2, COVID-19, SEIR model transmission dynamics, Mathematical modelling

## Abstract

**INTRODUCTION:**

Severe acute respiratory syndrome coronavirus 2 has been transmitted to more than 200 countries, with 92.5 million cases and 1,981,678 deaths.

**METHODS:**

This study applied a mathematical model to estimate the increase in the number of cases in São Paulo state, Brazil during four epidemic periods and the subsequent 300 days. We used different types of dynamic transmission models to measure the effects of social distancing interventions, based on local contact patterns. Specifically, we used a model that incorporated multiple transmission pathways and an environmental class that represented the pathogen concentration in the environmental reservoir and also considered the time that an individual may sustain a latent infection before becoming actively infectious. Thus, this model allowed us to show how the individual quarantine and active monitoring of contacts can influence the model parameters and change the rate of exposure of susceptible individuals to those who are infected.

**RESULTS:**

The estimated basic reproductive number, *R*_*o*_ , was 3.59 (95% confidence interval [CI]: 3.48 - 3.72). The mathematical model data prediction coincided with the real data mainly when the social distancing measures were respected. However, a lack of social distancing measures caused a significant increase in the number of infected individuals. Thus, if social distancing measures are not respected, we estimated a difference of at least 100,000 cases over the next 300 days.

**CONCLUSIONS:**

Although the predictive capacity of this model was limited by the accuracy of the available data, our results showed that social distancing is currently the best non-pharmacological measure.

## INTRODUCTION

Severe acute respiratory syndrome coronavirus 2 (SARS-CoV-2) belongs to the family *Coronaviridae* ^1^ ^,^ ^2^. SARS-CoV-2 has been transmitted to more than 200 countries with 96.0 million cases and 2,049,232 deaths worldwide^3^ ^,^ ^4^. The coronavirus disease (COVID-19) has devastated health, economic, and social infrastructures worldwide and is considered the largest pandemic crisis of the 21st century. SARS-CoV-2 emerged in Wuhan, China, in December 2019. The local epidemic rapidly spread to multiple countries, with consequent challenges for surveillance and control^5^. The first case of SARS-CoV-2 infection in Brazil was confirmed on February 26, 2020, in São Paulo (SP), the 8th largest city in the world, with 12 million inhabitants^6^.

No treatment is available to date, and vaccines are not expected to be sufficiently widely available to control the SARS-CoV-2 pandemic within the coming year. The only current approaches to reduce the number of new cases and the transmission rate during this pandemic are those of classical epidemic control, including case isolation, contact tracing and quarantine, physical distancing, and hygiene measures^7^. Additionally, knowledge of the propagation pattern of COVID-19 and the prediction of the time evolution is of great importance to save lives and reduce the social and economic consequences of the disease^8^. These data can be incorporated by mathematical models to understand how SARS-CoV-2 spreads within a population.

Since SARS-CoV-2 transmission started in Wuhan, China, mathematical modeling has been at the forefront of shaping the decisions regarding non-pharmaceutical interventions to confine its spread worldwide^9^ ^,^ ^10^. The viral spread can be determined by observing the period of incubation (the period during which an infected individual shows nonspecific or early symptoms during the prodromal phase, before classical clinical symptoms) and can be represented by the susceptible exposed infected recovered (SEIR) model to evaluate how social measures of isolation and quarantine can alter mortality rates and the number of cases of infected individuals over time. Another factor to consider is the basic reproduction number (*R*_0_)**,** used to measure the potential transmission of a disease^11^.

The SEIR-A mathematical model proposed by Yang and Wang^12^ has been used to study the dynamic spread of SARS-CoV-2 in Wuhan, China. We adapted this model and applied it in SP state, Brazil. Parameters such as SARS-CoV-2 surface stability and environment-human and human-human routes were considered to demonstrate how quarantine and social distancing can help in controlling the pandemic. Likewise, the lack of these non-pharmaceutical interventions can increase the spread of SARS-CoV-2 and prolong the pandemic period in Brazil.

## METHODS

### Mathematical Modeling

**FIGURE 1:**
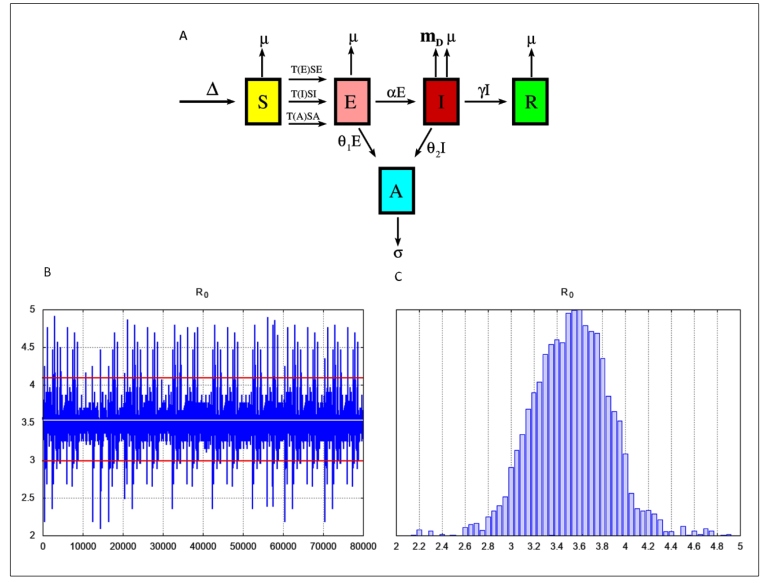
**(A):** Diagram of the SEIR-A model applied in the study to simulate SARS-CoV-2 spread. Each class is represented by its acronym: the susceptible population (*S*) is exposed to infection by direct and environmental transmission. In the exposed state (*E*), the population becomes infected (*I*). Infected individuals either die because of COVID-19 or recover (*R*). The exposed and infected populations spread the virus in environments (*A*) that can infect susceptible individuals. Δ: birth rate of the local population; μ: natural death rate;*T* (*E*)*SE*: constant transmission between susceptible and exposed individuals; *T* (*I*)*SI*: constant transmission between susceptible and infected individuals; *T* (*A*)*SA*: constant transmission between susceptible individuals and the environmental reservoir; θ_1_: rate of SARS-CoV-2 shedding by exposed individuals; α: incubation period between infection and the onset of disease symptoms; σ: rate of SARS-CoV-2 removal from the environment; *m*_*D*_: disease-related death rate; θ_2_: rate of SARS-CoV-2 shedding by infected individuals; γ: rate of COVID-19 recovery. **(B):** Trace plot output of *R*_*O*_ . **(C):** Histogram generated by the MCMC method for parameter *R*_*O*_ .

The mathematical model to describe the SARS-CoV-2 transmission in SP state divided the entire population into five classes: susceptible (*S*), exposed (*E*), infected (*I*), recovered (*R*), and environmental reservoir (*A*) class. The infected and exposed populations (individuals in the incubation period) can infect the susceptible population. Recovered individuals were those who were cured or who died of COVID-19. Finally, class *A* represented the indirect, environment-to-human transmission rate. SARS-CoV-2 spread among these classes and its circulation are represented in Figure 1.

Membership in the classes changes over time and one can conceptualize the time course of a pandemic as a movement of hosts among classes. Thus, the diagram shown in Figure 1 leads to the following system of ordinary differential (*d*) equations. Each set of dependent variables counts individuals in each of the groups, each as a function of time (*t*):

$$\frac{dS}{dt}=\;∆-T_{E}\left(E\right)SE-T_{I}\left(I\right)SI-T_{A}\left(A\right)SA-\;\mu S\;$$

$$\frac{dE}{dt}=\;T_{E}\left(E\right)SE+\;T_{I}\left(I\right)SI+\;T_{A}\left(A\right)SA-\left(\alpha +\;\mu \right)S$$

$$\frac{dl}{dt}=\;\alpha E-\left(m_{D}+\gamma +\;\mu \right)I$$ (2.1)

$$\frac{dR}{dt}=\;\gamma I-\;\mu R$$

$$\frac{dA}{dt}=\;\theta_{1}E+\;\theta_{2}I-\;\sigma A,$$

where Δ is the birth rate of the local population; *T*_*E0*_ is constant transmission between susceptible and exposed individuals [*ET*(*E*)*SE*]; *T*_*A0*_ is constant transmission between susceptible and infected individuals [*T* (*I*)*SI*]; *T*_*A0*_ is constant transmission between susceptible and environmental reservoir [*T*(*A*)*SA*]; μ is natural death rate; α is the incubation period between infection and the onset of disease symptoms; *m*_*D*_ is the disease-related death rate; γ is the recovery rate for the COVID-19; θ_1_ is SARS-CoV-2 shedding rate by exposed individuals; θ_2_ is the rate of SARS-CoV-2 shedding by infected individuals; and σ is the rate of SARS-CoV-2 removal from the environment.

The functions *T*_*E*_ (*E*) and *T*_*I*_ (*I*) represent human-to-human transmission rates between exposed and susceptible and between infected and susceptible individuals, respectively, and require adjustment for the transmission coefficient (*c*), which in this study was given by:

$$T_{E}\left(E\right)=\;\frac{T_{E0}}{1+cE\;}\;\;\;and\;\;\;T_{I}\left(I\right)=\;\frac{T_{I0}}{1+cI\;}$$ (2.2)

where *T*_*E0*_ and *T*_*I0*_ express the maximum transmission rates. The function *T*_*A*_ (*A*) represents the environmental-to-human transmission rate and is given by:

$$T_{A}\left(A\right)=\;\frac{T_{A0}}{1+cA}$$ (2.3)

The basic reproduction number *R*_0_ is defined as the expected number of secondary cases produced by a single (typical) infection in a completely susceptible population^13^. The model used in this study defined *R*_0_ as:

$$R_{0}=\;\frac{T_{E}(0)S_{0}}{\alpha +\;\mu }+\;\frac{{\alpha T}_{I}(0)S_{0}}{\omega_{1}(\alpha +\;\mu )}+\;\frac{{(\omega_{1}\theta }_{1}+\;\alpha \theta_{2})T_{A}(0)S_{0}}{\sigma \omega_{1}(\alpha +\;\mu )}\;=\;R_{1}+\;R_{2}+\;R_{3}$$ (2.4)

where, *S*_0_ is the initial percentage of the susceptible population and ω_1_ is the sum of *m*_*D*_ , α and μ parameters. Thus, *R*_O_ = 1 is a threshold parameter to quantify SARS-CoV-2 spread by estimating the average number of secondary infections in a wholly susceptible population. If *R*_O_ < 1, the number of infected individuals decreases over time as SARS-CoV-2 is contained. However, if, the number of infected individuals increases and SARS-CoV-2 persists. The term *R*_1_ measures the contribution from exposed to susceptible individuals’ transmission, while *R*_2_ measures the contribution from infected to susceptible individuals’ transmission. The third term, *R*_3_ , represents the contribution from the environmental-to-human transmission route. These three transmission modes collectively shape the overall infection risk for the SARS-CoV-2 pandemic.

## RESULTS

### Parameter estimation and model fitting

The numerical validation and computational simulations of the mathematical model proposed by the system of equations (2.1) used cumulative reported data from the COVID-19 daily bulletin from the SP city Health Department that has statewide data^14^. The data were based on confirmed testing between February 25 and July 05, 2020, with 320,179 confirmed infections.

The mathematical model proposed by the system of equations (2.1) was implemented in the mathematical software Octave and numerical simulations were performed for an epidemic period between February 25 and July 05, 2020. The estimated population for SP state is over 45 million^6^ and the state was placed under quarantine by the current governor on March 24, 2020. In the epidemic period, our simulations assumed that only a relatively “small” number of people have traveled to SP state; thus, the inflow rate (Δ) of the model is based only on the number of newborns in the state. Spencer et al.^15^ reported an average recovery period of approximately 15 days; hence, we defined the recovery rate from COVID-19 as γ = 1/15 per day. The incubation period of the infection varied between 2 and 14 days, with an average of 5-7; therefore, σ = 1/15 . Kampf et al.^16^ reported that some members of the *Coronaviridae* family can remain infectious in the environment from 2 hours up to 5-9 days. We considered several values for the σ parameter; namely, 0 < = σ < = 1, depending on the date of the computer simulation. The transmission rate (*T*_*E0*_ and *T*_*I0*_ ) values were estimated as described by Tang et al.^17^. Additionally, θ_1_ and θ_2_ were estimated using a Markov chain Monte Carlo (MCMC) method in our computer simulation (the MCMC method is described in Supplementary Material 1). On March 24, a strict policy of social distancing was implemented, with medical care offered to confirmed cases; thus, SARS-CoV-2 spread by infected individuals to the environment was considered low. Therefore, between March 24 and April 24, we considered θ_2_ = 0 and θ_1_ > 0 .

**FIGURE 2:**
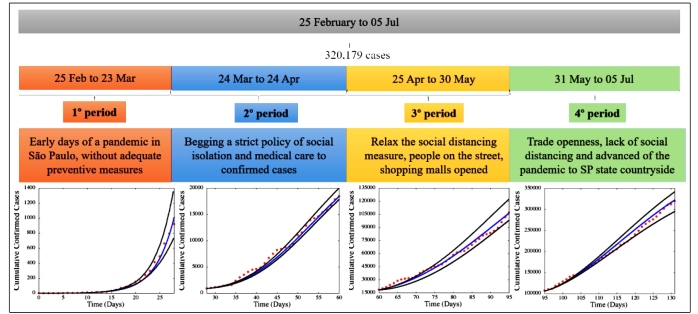
Cumulative confirmed cases in four different periods. In the graphs at the bottom of the figure, the solid blue line denotes the result of the computer simulation, the red balls denote the reported cases of COVID-19, and the solid black lines represent the lower and upper bounds of the 95% CI for all 10,000 simulations.

The present study also considered the presence of SARS-CoV-2 in the environment. For this, three parameters were determined: the adjustment coefficient (*c*), the rate (θ_1_), and environment-to-human constant transmission (*T*_*A0*_ ). To estimate the value of θ_1_ , we applied MCMC methods based on the adaptive combination Delayed rejection and Adaptive Metropolis (DRAM) algorithm^18^ ^,^ ^19^ to the system (2.1) (Supplementary Material 1 **)**. We sampled from 80,000 MCMC iterations and discarded the first 10,000 samples as a burn-in period. Based on these 70,000 samples, the point estimates and 95% confidence intervals (CIs) for those parameters were calculated. Based on the fitted model, the estimated *R*_0_ was 3.59 (95% CI: 3.48 - 3.72), which meant that each infected person could infect an average of 3.59 people during the infection period. Lastly, θ_1_ , *T*_*A0*_ and *c* values and 95% CIs were determined for the four epidemic periods analyzed and were similar to the *R*_0_ parameter^20^ ^,^ ^21^. The first conditions for the five classes of the differential equation system and parameter values used in the computational model for the four different simulation periods are shown in Table 1. Using the estimated parameter values, we assessed the fit between the model solution and real data, as shown in Figure 2.

**TABLE 1:** Initial conditions for the five classes of differential equation system and parameter values used in the computational model.

Parameters	First period	Std	Second period	Std	Third period	Std	Fourth period	Std	Source
*T*_A0_	4.04*x*10⁻¹⁰	4.41*x*10⁻¹¹	4.15*x*10⁻¹⁰	4.57*x*10⁻¹¹	1.03*x*10⁻¹¹	5.44*x*10⁻¹²	1.12*x*10⁻¹¹	6.15*x*10⁻¹²	This study
*c*	4.03*x*10⁻⁵	6.17*x*10⁻⁶	4.93*x*10⁻⁵	6.93*x*10⁻⁶	3.71*x*10⁻⁶	9.81*x*10⁻⁷	1.27*x*10⁻⁶	7.38*x*10⁻⁷	This study
θ_1_	2.376	0.426	3.786	0.535	2.135	0.394	4.052	0.547	This study
*S* (0)	45,919,049	-----	45,907,329	-----	45,854,629	-----	45,511,907	-----	^22^
*E* (0)	1	-----	800	-----	15,000	-----	100,000	-----	^14^
*I* (0)	1	-----	820	-----	18,420	-----	107,142	-----	^14^
*R* (0)	0	-----	100	-----	1,000	-----	100,000	-----	^14^
*A* (0)	6	-----	10,000	-----	30,000	-----	100,000	-----	^14^
*T*_E0_	6.32*x*10⁻⁹	7.11*x*10⁻¹⁰	6.02*x*10⁻⁹	6.83*x*10⁻¹⁰	5.02*x*10⁻⁹	4.14*x*10⁻¹⁰	4.52*x*10⁻⁹	3.38*x*10⁻¹⁰	^17^
*T*_*I0*_	3.32*x*10⁻⁹	7.92*x*10⁻¹⁰	1.22*x*10⁻⁹	6.67*x*10⁻¹⁰	1.01*x*10⁻⁹	6.04*x*10⁻¹⁰	7.61*x*10⁻¹⁰	4.74*x*10⁻¹¹	^17^
θ_2_	1.037	0.373	0.00	-----	1.247	0.389	0.863	0.291	This study
Δ	1,659.26	-----	1,659.26	-----	1,659.26	-----	1,659.26	-----	^22^
*m*_*D*_	0.0372/day	-----	0.045/day	-----	0.05/day	-----	0.05/day	-----	^14^
μ	3.5*x*10^-5^/day	-----	3.5*x*10^-5^/day	-----	3.5*x*10^-5^/day	-----	3.5*x*10^-5^/day	-----	^22^
α	5 days	-----	5 days	-----	5 days	-----	5 days	-----	^15^
γ	1/15/day	-----	1/15/day	-----	1/15/day	-----	1/15/day	-----	^15^
σ	0.2/day	-----	1/day	-----	0.2/day	-----	0.2/day	-----	^23^

*T*_*A0*_ **:** constant transmission between susceptible individuals and the environmental reservoir; *c:* transmission coefficient; θ_1_: rate of SARS-CoV-2 shedding by exposed individuals; *S* (0): susceptible individuals; *E* (0): exposed individuals; *I* (0): infected individuals; *R* (0): recovered individuals; *A* (0): environmental reservoir; *T*_*E 0*_: constant transmission between susceptible and exposed individuals; *T*_*I 0*_: constant transmission between susceptible and infected individuals; θ_2_: rate of SARS-CoV-2 shedding by infected individuals; Δ: birth rate of the local population; *m*_*D*_: disease-related death rate; μ: natural death rate; α: incubation period between infection and the onset of disease symptoms; γ: recovery rate from COVID-19; σ: rate of SARS-CoV-2 removal from the environment

### Numerical results

To illustrate the estimated *R*_0_ before the quarantine (the first period), Figure 1b shows a trace plot of the MCMC output using 80,000 MCMC samples. The histograms of *R*_0_ values generated by the MCMC method are shown in Figure 1c. 

The estimated *R*_0_ was 3.59 before the quarantine (first period). For the second, third, and fourth periods, we instead estimated the effective reproductive number (*R*_t_ ). The estimated *R*_t_ values were 1.972 (95% CI: 1.535 - 2.427), 1.753 (95% CI: 1.253 - 2.239) and 1.558 (95% CI: 0.973 - 1.879) in the second, third, and fourth periods, respectively. The numbers of cumulative confirmed cases for the four epidemic periods of COVID-19 in SP state versus the adjustment curves are shown in Figure 2. We observed a good fit between the model solution and real data with 95% CIs for all 10,000 simulations. The good agreement between solutions validated our results.

**FIGURE 3:**
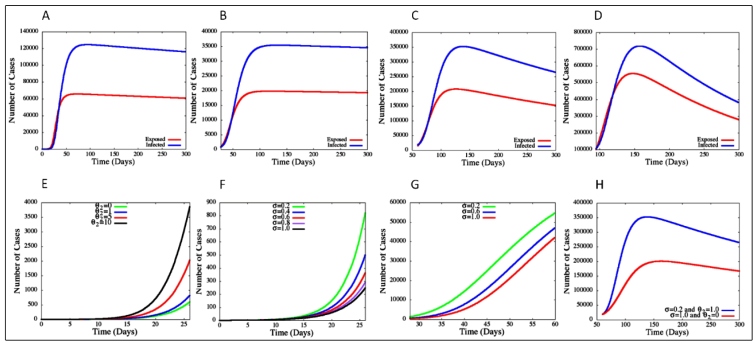
Results of numerical simulations to predict the cumulative number of SARS-CoV-2 infected and exposed individuals in SP state during four different time periods (**A** to **D)**, as well as the effects of the rate of SARS-CoV-2 removal from the environment in SP state among confirmed cases of infection. **(E):** First period and θ_2_ = 1. **(F):** Second period and θ_2_ = 0. **(G):** Match effects of the policy of social distancing (θ_2_) and the removal rate of SARS-CoV-2 (σ) from the environment in SP state from confirmed cases in the first period. (**H**): Projection of individuals infected between April 25, 2020, and February 19, 2021 (300 days later).

We used a computational mathematical model to determine the trend in the numbers of cumulative cases of infected and exposed individuals (Figure 3). The numerical simulation to the first period showed that the infection level increased up to 90-100 days (Figure 3A), peaking at around 124,000 infected individuals on June 4, 2020. In the second period, with a policy of maintaining social distancing, the numerical simulation showed that the infection level increased up to 65-70 days, peaking at approximately 36,000 infected individuals on June 2, 2020 (Figure 3B). During the third period, with the relaxing of social distancing measures, the infection level increased up to 80-90 days, peaking at approximately 352,500 infected individuals on July 25, 2020 (Figure 3C). Finally, in the fourth period, with trade openness, lack of social distancing, and advancing of the pandemic to the SP countryside, the infection level increased up to 60-70 days, peaking at approximately 718,610 infected individuals on July 05, 2020 (Figure 3D).

### **Variations in *θ***_2_

The θ_2_ value increased when there was a reduction in social distancing, reflecting the number of individuals infected by SARS-CoV-2 (Table 2). Variations in the numbers of confirmed cases for different θ_2_ values are shown in Figure 3E. When θ_2_ = 0, the contribution of infected individuals, like the SARS-CoV-2 environmental reservoir, is low. The predicted number of cases on March 23 was 779, a value below the actual number of confirmed cases (860). When θ_2_ = 1, about 18,265 cases were predicted for April 24, a number that differed slightly from the actual number of confirmed cases (17,826). However, when θ_2_ = 10, the model predicted 4,830 cases on March 23, different from the actual number of confirmed cases (860). 

**TABLE 2:** Predictions of confirmed cases for σ = 0.2 (1^o^ period) and σ = 1 (2^o^ period) with different values of *θ*_2_ parameters.

Date	25/02	01/03	06/03	12/03	17/03	20/03	23/03	25/03	30/03	05/04	11/04	16/04	20/04	24/04
Predicted confirmed cases θ_2_ = 0	1	2	7	38	153	349	779	860	1,657	4,661	8,057	11,132	14,276	17,840
Predicted confirmed cases θ_2_ = 1	1	2	8	46	174	411	890	860	1,680	4,781	8,251	11,391	14,541	18,265
Predicted confirmed cases θ_2_ = 5	1	2	12	92	473	1,171	2,617	862	1,757	5,142	8,813	12,057	15,255	18,890
Predicted confirmed cases θ_2_ = 10	1	3	18	174	981	2,347	4,830	863	1,827	5,437	9,245	12,550	15,765	19,366
Real data of confirmed cases	1	1	10	42	164	413	860	862	1,537	4,620	8,216	11,043	14,267	17,826

σ*:* rate of SARS-CoV-2 removal from the environment; **1** ^o^ **period**: February 25 to March 23, 2020; **2** ^o^ **period**: March 24 to April 24, 2020; θ_2_: rate of SARS-CoV-2 shedding by infected individuals.

### Variations in σ

The *σ* parameter in the SEIR-A model indicates the rate of SARS-CoV-2 removal from the environment. Variations in the confirmed numbers of cases for different σ values are shown in Figure 3. The effects of SARS-CoV-2 removal rate in the first period, when σ = 0.2 (green line in Figure 3F) suggested that approximately 5 days were required to decrease SARS-CoV-2 in the environment^12^. During this period, the number of cases predicted by our model (890) was consistent with the actual number of confirmed cases (830). A removal rate (σ) of 1 suggested that approximately 1 day was required to decrease SARS-CoV-2 circulation in the environment^12^, with 314 predicted infections, a number smaller than the actual number of confirmed cases. In the second period (θ_2_ = 0 and σ = 1), there were 17,840 predicted infections on April 24 (red line on Figure 3G), very close to the actual number of confirmed cases (17,826).

The effects of the social distancing policy (measured by θ_2_ ) and the rate of SARS-CoV-2 removal from the environment (measured by σ) are shown in Figure 3H (red line) from the time of the initial implementation of the strict social distancing, indicating the projected number of people infected between April 25, 2020, and February 19, 2021 (300 days later). The results of our model showed that maintaining non-therapeutic measures resulted in 170,000 rather than 270,000 cases’.

## DISCUSSION

This study applied an SEIR-A model that considered the potential routes from the reservoir to a person and from person to person of SARS-CoV-2, respectively, to compare the estimated data to the reported data for four epidemic periods of COVID-19 in SP state, Brazil. All scenarios showed agreements between the numerical solutions obtained via the mathematical model and the actual data on the number of confirmed cases. Moreover, the SEIR-A model was also used to predict SARS-CoV-2 spread in SP state for the next 300 days. 

The model incorporated multiple transmission pathways as well as an environmental class that represented the pathogen concentration in the environmental reservoir. Here, the term "environmental reservoir" refers to the presence of SARS-CoV-2 in urban areas based on findings reported by Abrahão et al.^24^ regarding the detection of SARS-CoV-2 RNA on public surfaces in a densely populated urban area in Brazil. Using sterile swabs, the authors evaluated 101 samples collected from different surfaces near the hospital and public transportation sites and submitted them for nucleic acid extraction and genomic detection and quantification by one-step quantitative polymerase chain reaction (qPCR). Seventeen (16.8%) samples collected from bus stations, public squares, and sidewalks tested positive for SARS-CoV-2 RNA, including samples obtained near hospitals. Thus, the study results demonstrated the contamination of public surfaces by SARS-CoV-2, especially near hospital areas, highlighting the risk of infection. Additionally, the US Centers for Disease Control and Prevention (CDC)^25^ also recognizes the risk for individuals to be infected by SARS-CoV-2 by touching a surface or object contaminated with the virus and then touching their mouths, noses, or eyes. While this is not thought to be the main route of viral spread, we are still learning more about how this virus spreads.

To better understand how the virus spreads among people and via objects, Böhmer et al.^26^ studied the transmission of SARS-CoV-2 from patient 0 (a Chinese resident who visited Germany for professional reasons) until the infection of patient 16. The infection of patient 5 by patient 4 happened in a single encounter during a canteen visit, with the patients sitting back-to-back, when patient 5 borrowed a saltshaker from patient 4, thus demonstrating the potential for contamination via objects. Thus, the environment acts as a reservoir for SARS-CoV-2 and can lead to the infection of susceptible individuals.

To prevent individual and community transmission, an accurate test for SARS-CoV-2 and appropriate preventive measures are paramount^27^. As the epidemic progresses, all tools available for SARS-CoV-2 diagnosis must be applied. COVID-19 daily bulletin data from SP city Health Department^14^ contains the results of reverse transcription-qPCR (RT-qPCR), rapid tests for antibody and antigen detection, enzyme-linked immunosorbent assay (ELISA) tests, and other types of tests. While RT-qPCR detects active SARS-CoV-2 infection, serological tests based on immunoglobulin G (IgG) show previous exposure to SARS-CoV-2. These differences impact our estimates, especially the numbers of infected individuals. However, the underreporting of cases and high percentages of asymptomatic and pre-asymptomatic individuals also contribute to the spread of SARS-CoV-2^28^. Thus, the data generated in our study should be used with caution.

The basic reproduction number *R*_0_ is a powerful quantitative concept used to characterize the contagiousness and transmissibility of SARS-CoV-2^29^ ^,^ ^30^. This number reflects how new infections are caused by a single infectious individual in an otherwise completely susceptible population^30^ ^,^ ^31^. The *R*_0_ in all scenarios in our simulations was > 1 (3.59 to 1.558), with greater values observed when no measures had been implemented to prevent virus spread, as occurred in Wuhan, China^32^. *R*_0_ > 1 indicated the highest number of infected people and the consequent persistence of SARS-CoV-2 in SP state. 

Comparison of the results obtained in the numerical simulations to real data from the confirmed cases showed that the mathematical modeling satisfactorily predicted the cases that occurred in the first period (February 25 to March 23, 2020) (Table 2). In particular, the predictions on March 20 and 23, 2020 were approximately 411 and 890 cases, nearly identical to the number of confirmed cases on those dates (413 and 860). During the second period, approximately 14,276 and 17,840 cases were predicted for April 20 and 24, 2020 were, respectively, also very close to the actual number of confirmed cases of 14,267 and 17,826. However, the discrepancy observed between the predicted and confirmed cases was directly related to the relaxation of social distancing measures. Because of the greater number of infected people, the virus spread in the environment^33^. 

In contrast, the removal of SARS-CoV-2 from the environment decreases the number of confirmed infected cases according to the increase in σ. Thus, measures like hospitalization or isolation of individuals with positive diagnoses, tracking of new cases, and strict isolation to reduce contact with infected individuals will increase the rate of removal of SARS-CoV-2 from the environment, reflecting a smaller number of cases (100,000 fewer cases over the next 300 days). Respiratory infectious diseases, such as those caused by SARS-CoV-2, are spread through a susceptible individual’s contact with the virus. These contacts facilitate disease transmission and can be made indirectly through environmental routes or direct person-to-person interactions^33^. Thus, measures such as wearing masks, social distancing, isolation of positive cases, and tracking of new cases are essential to mitigating the COVID-19 pandemic in SP state, Brazil and, therefore, must be enforced by the government in the form of law.

We emphasize that the mathematical model has limitations. We used official data from the State Health Secretariats, which releases data after some days of delay. It is important to consider that, in Brazil, people hospitalized or who come to the hospital with flu-like signs, and sometimes, contacts of positive patients, are tested for SARS-CoV-2 infection. Thus, the number of cases considered positive may be higher than the reported cases, which does not invalidate our results because the most significant population in this study was patients requiring medical care, who can lead to the collapse of the public health system. Therefore, the results of in study can be used to evaluate the effects of a strict policy of social isolation, preventive measures, and decisions for new strategies to reduce the SARS-CoV-2 pandemic.

In conclusion, we used a mathematical model to show the effects of social distancing on the number of cases of SARS-CoV-2 infection during the pandemic in SP state. We showed that the discrepancy observed between the predicted and confirmed numbers of cases was directly related to the relaxation of social distancing measures. Therefore, the duration of social distancing has significantly decreased the number of infected people in SP state. Our model showed that maintaining non-therapeutic measures resulted in 170,000 rather than 270,000 cases at the end of 300 days. Thus, if we do not have a SARS-CoV-2 vaccine, we believe that non-therapeutic measures are the best strategy to combat the disease.
