# Supplementary material for: Modeling transmission dynamics of severe acute respiratory syndrome coronavirus 2 in São Paulo, Brazil
Source: Rev Soc Bras Med Trop. 2021 Jan 29;54:e05532020. doi: 10.1590/0037-8682-0553-2020 (PMC7849330; doi:10.1590/0037-8682-0553-2020)
Supplement: Supplementary file 1 [file 1678-9849-rsbmt-54-e05532020-suppl1.pdf]

Supplementary material 1 – Estimation of values for each parameter and description of the MCMC method

1) S_0 (susceptible individuals): data obtained from the SEADE homepage at https://www.seade.gov.br/2020/ (reference 22).

2) E_0, I_0, R_0, and A_0 (exposed, infected, and recovered individuals and the environmental reservoir, respectively): data obtained from the Boletins COVID-19 homepage at https://www.prefeitura.sp.gov.br/cidade/secretarias/saude/vigilancia_em_saude/doencas_e_agravos/coronavirus/index.php?p=295572 (reference 14).

3) α (incubation period between infection and the onset of disease symptoms) and γ (COVID-19 recovery rate): data obtained from Spencer et al., 2020 (reference 15).

4) $\mu\ (natural\ death\ rate) = 3.5\ \times\ 10^{-5} = 1/(78.4\ \times 365)$, given an average life expectancy of 78.4 years in the state of SP. Data obtained from the SEADE homepage at https://www.seade.gov.br/2020/ (reference 22).

5) Δ (birth rate of the local population) = 1,659.26. Data obtained from the SEADE homepage at https://www.seade.gov.br/2020/ (reference 22).

6) m_D (disease-related death rate): Data obtained from the Institute for Health Metrics and Evaluation homepage: http://www.healthdata.org/brazil-s%C3%A3o-paulo and from the Boletins COVID-19 in homepage at https://www.prefeitura.sp.gov.br/cidade/secretarias/saude/vigilancia_em_saude/doencas_e_agravos/coronavirus/index.php?p=295572 (reference 14).

7) σ (rate of SARS-CoV-2 removal from the environment): According to Geller et al., 2012 (reference 23) the duration of the virus in the environment can vary from 1 day to 5–9 days; thus, we calculated the SARS-CoV-2 removal rate as $\sigma = 1/1\,day = 1$ for the 2nd period (higher proportion of social distancing, according to Yang & Wang, 2020 (reference 12). For periods of low adherence to social distancing, we used a virus removal rate of $\sigma = 1/5\,day = 0.2$.

8) θ_2 (rate of SARS-CoV-2 shedding by infected individuals): We used $\theta_2 = 0$ for the 2nd period (higher proportion of social distancing and quarantine for isolated cases, according to Yang & Wang, 2020 - reference 12). For periods of low adherence to social distancing, we determined θ_2 using the Markov chain Monte-Carlo (MCMC) method described below.

9) We estimated the unknown parameters based on the total number of confirmed cases by using extensive Markov chain Monte-Carlo (MCMC) simulations based on the adaptive combination Delayed rejection and Adaptive Metropolis (DRAM) algorithm for the system (equation 2.1 in the manuscript).

9.1) The total number of confirmed cases can be expressed as:

$$dC_c/dt = \theta_1 e + \theta_2 I,$$

where $C_c(t)$ is the total number of confirmed cases.

9.2) The total number of confirmed new cases can be expressed as:

$$NC_c(t) = C_c(t) - C_c(t-1) \dots\dots\dots\dots\dots\dots (1),$$

where $NC_c(t)$ represents the total number of confirmed new cases.

9.3) The Markov chain Monte-Carlo (MCMC) method was used to fit the equation (1) and, thus, estimate the other parameters. For example, we used 80,000 sample realizations to acquire the parameter value for θ_1 (rate of SARS-CoV-2 shedding exposed individuals) with the MCMC chain in Figure 1 below.

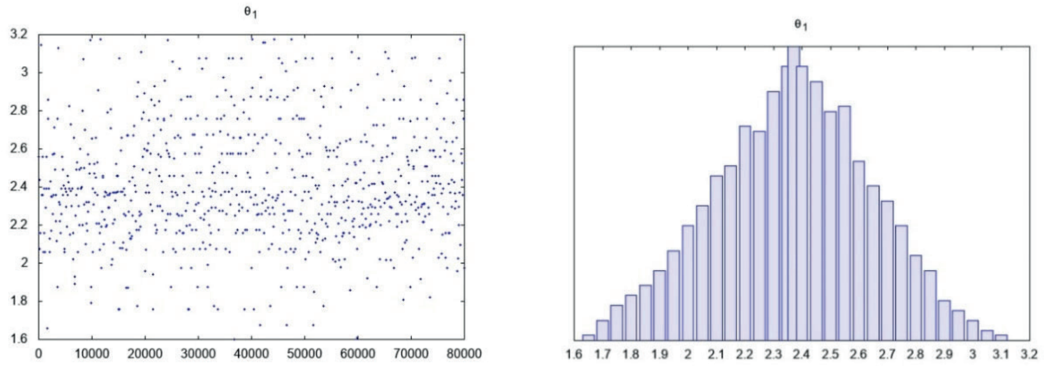

(a) (b)

FIGURE 1: (a) Simulation results for parameter θ_1, from February 25, 2020, to March 23, 2020, of the MCMC chain with 80,000 sample realizations. (b) Histogram of the MCMC chain for parameter θ_1. θ_1: Rate of SARS-CoV-2 shedding by exposed individuals.

9.4) The mean value, standard deviation, and MCMC error for the σ parameter as well as their 95% confidence intervals are presented in Table 1.

TABLE 1: Parameter estimation for parameter θ_1 using the MCMC method for cumulative daily reported case data from February 25, 2020, to March 23, 2020.

Parameter	Mean Value	Standard Deviation	MCMC Error
θ_1	2.376	0.4257	1.5382e-04
